# Evaluating the Association of Calcified Neurocysticercosis and Mesial Temporal Lobe Epilepsy With Hippocampal Sclerosis in a Large Cohort of Patients With Epilepsy

**DOI:** 10.3389/fneur.2021.769356

**Published:** 2022-01-27

**Authors:** Thaís Leite Secchi, Rosane Brondani, José Augusto Bragatti, Jorge Wladimir Junqueira Bizzi, Marino Muxfeldt Bianchin

**Affiliations:** ^1^Graduate Program in Medicine: Medical Sciences, Universidade Federal Do Rio Grande Do Sul, Porto Alegre, Brazil; ^2^Division of Neurology, Hospital de Clínicas de Porto Alegre, Porto Alegre, Brazil; ^3^CETER—Center for Epilepsy Surgery, Hospital de Clínicas de Porto Alegre, Porto Alegre, Brazil; ^4^Division of Neurosurgery, Hospital de Clínicas de Porto Alegre, Porto Alegre, Brazil; ^5^Basic Research and Advanced Investigations in Neurology, Hospital de Clinicas de Porto Alegre, Universidade Federal Do Rio Grande Do Sul, Porto Alegre, Brazil

**Keywords:** epileptogenesis, initial precipitating injury (IPI), hippocampal sclerosis, inflammation in epilepsy, gender differences in epilepsy, neurocysticercosis

## Abstract

**Background:**

Neurocysticercosis (NCC) is a parasitic infection of the central nervous system that has been associated with mesial temporal lobe epilepsy with hippocampal sclerosis (MTLE-HS). However, this association has not been completely established.

**Objective:**

To evaluate the prevalence of calcified NCC (cNCC), its characteristics and a possible association between cNCC and MTLE-HS in a cohort of 731 patients with epilepsy.

**Methods:**

We review clinical, EEG and neuroimaging findings of 731 patients with epilepsy. From these, 659 had CT-scans and 441 patients had complete neuroimaging with CT-scans and MRI. In these patients, we review the prevalence and characteristic of epilepsy in cNCC and in MTLE-HS patients.

**Results:**

Forty-two (6.4%) of the 659 patients studied with CT-scans had cNCC. cNCC lesions were more frequent in women than in men (*n* = 33–78.6% vs. *n* = 09–21.4%, respectively; OR = 3.64;(95%CI = 1.71–7.69); *p* < 0.001). cNCC was more often in patients who developed epilepsy later in life, in older patients, in patients who had a longer history of epilepsy, and in those with a lower educational level. MTLE–HS was observed in 93 (21.1%) of 441 patients that had complete neuroimaging, and 25 (26.9%) of these 93 patients also had cNCC. Calcified NCC was observed in only 17 (4.9%) of the remaining 348 patients that had other types of epilepsy rather than MTLE-HS. Thus, in our cohort, cNCC was more frequently associated with MTLE-HS than with other forms of epilepsy, O.R. = 11.90;(95%CI = 6.10–23.26); *p* < 0.0001).

**Conclusions:**

As expected, in some patients the epilepsy was directly related to cNCC lesional zone, although this was observed in a surprisingly lower number of patients. Also, cNCC lesions were observed in other forms of epilepsy, a finding that could occur only by chance, with epilepsy probably being not related to cNCC at all. In this cohort, cNCC was very commonly associated with MTLE-HS, an observation in agreement with the hypothesis that NCC can contribute to or directly cause MTLE-HS in many patients. Given the broad world prevalence of NCC and the relatively few studies in this field, our findings add more data suggesting a possible and intriguing frequent interplay between NCC and MTLE-HS, two of the most common causes of focal epilepsy worldwide.

## Introduction

Neurocysticercosis (NCC) is a human infection caused by *Taenia solium*. It is one of the most common parasitic infections of the central nervous system (CNS), affecting millions of people worldwide (1–5). The disease is endemic in Latin America, Asia and Africa and has also been reported in developed countries (6–11). One of the main symptoms of NCC is epilepsy. NCC has been associated with seizures during active infection, when cysticerci are degenerating, or when NCC becomes a calcified neurocysticercosis (cNCC) lesion (12–19). The epileptogenic mechanisms involved in the epilepsy provoked by NCC have been extensively studied, but they are still not completely elucidated (12–19). Furthermore, the treatments and prognosis of epilepsy associated with NCC have not been entirely established (20–22).

Mesial temporal lobe epilepsy associated with hippocampal sclerosis (MTLE-HS) is one of the most common types of focal epilepsy in humans and a form of epilepsy that is often resistant to pharmacological treatment (23–26). Briefly, in this form of epilepsy, patients usually develop hippocampal damage after some serious neurological insult at an early age (23–26). The event associated with hippocampal damage usually occurs during childhood and is known as initial precipitating injury (IPI) (27–32). Several different events can be considered IPIs, among them traumatic brain injury, prolonged febrile seizures and *status epilepticus* (27–32). Some forms of acute CNS infections can also cause hippocampal damage and therefore are also potential forms of IPIs and possibly causes of MTLE-HS (27–32). NCC is a form of CNS parasitic infection and some authors have associated it with hippocampal damage or development of MTLE-HS (33–45). However, these studies are relatively recent in the history of epilepsy and NCC and were done at few centers, mostly in epilepsy surgery centers, where drug-resistant epileptic patients are treated. Thus, these studies might be susceptible to selection or interpretation bias and their conclusions may have several limitations. One way to improve these biases is to perform studies in large cohorts of patients selected from the community and not only from the surgical epilepsy centers.

In the present study, an entire cohort of 731 patients that have been followed for a long time at our outpatient epilepsy clinic was selected. From these, 659 had CT-scans and 441 patients had complete neuroimaging evaluations with CT-scan and MRI. Data of these patients were reviewed for the prevalence of cNCC, its characteristics and its possible association with MTLE-HS. We studied the entire cohort to better understand the possible impact of the NCC in MTLE-HS. It is possible that these data might eventually help to expand the understanding of the relevance and impact of a possible association between NCC and MTLE-HS, two of the most common causes of focal epilepsy worldwide.

## Methods

The charts of a cohort of all 731 patients with a diagnosis of epilepsy who were being followed at the epilepsy outpatient clinic of Hospital de Clínicas de Porto Alegre (HCPA) were reviewed. These patients are the entire cohort of patients that are followed in our service. All of these patients are seen at least once a year and, thus, all patients with a diagnosis of epilepsy in the out-patient epilepsy clinic were included in this study. Brazil is a large and heterogeneous country. Its territorial extension is 8,516,000 km^2^, divided into 26 states (46). Although the WHO considers Brazil to be a country where NCC is endemic, the disease is common in some regions, but is becoming much less common in others. HCPA is a public university institution, part of the network of university hospitals of the Brazilian Ministry of Education and is academically linked to the Federal University of Rio Grande do Sul (UFRGS). HCPA is supported by Sistema Unico de Saude (SUS), the unified public health system of Brazil, and one of the largest public health systems in the world. HCPA is certified by the JOINT Commission and ranks 7th in the Latin American ranking of best hospitals (47). HCPA is located in Porto Alegre, the capital of the state of Rio Grande do Sul, southern Brazil, where most patients in this study reside. The city has a population of 1,416,735 inhabitants, distributed over an area of 496.8 km^2^ and is the fourth capital with the highest Human Development Index in the country (48).

Clinical, electrophysiological and neuroimaging data were evaluated. Inclusion criteria were: having an established diagnosis of epilepsy according to the International League Against Epilepsy (ILAE) (49, 50); being followed up for at least one year (or more) at our epilepsy clinic; and being older than 15 years. All CT scans that were performed in most patients were reviewed because they are important for detecting cNCC lesions. Exclusion criteria were having a diagnosis other than epilepsy. For most analyses, patients were divided into two groups: (a) epilepsy with cNCC and (b) all other epilepsies. Definitive neurocysticercosis was diagnosed if the following features were present: (i) an absolute criterion such as histological demonstration of the parasite or cystic lesions showing the scolex on CT or MRI; (ii) two major criteria, such as lesions highly suggestive of neurocysticercosis in neuroimaging studies, spontaneously resolving small single enhancement lesions, or resolution of intracranial cystic lesions after albendazole or praziquantel therapy; or (iii) one major and two minor criteria such as lesions compatible with neurocysticercosis on neuroimaging studies, clinical manifestations suggestive of neurocysticercosis, and positive cerebrospinal fluid (CSF) ELISA for the detection of anticysticercosis antibodies, in addition to epidemiological evidence. According to the above criteria, the presence of dense, solid supratentorial calcifications, 1–10 mm in diameter, in the absence of other disease, should be considered highly suggestive of neurocysticercosis in areas endemic for NCC, as is the case of our country. Probable neurocysticercosis was diagnosed if the following features were present: (i) one major and two minor criteria; (ii) one major criterion, one minor criterion and epidemiological evidence, and (iii) three minor criteria and epidemiological evidence (51–55).

In this study, diagnostic criteria for neurocysticercosis was based in recent recommendations for neurocysticercosis diagnosis (55). All patients included in this study with diagnostic of neurocysticercosis had at least one major neuroimaging criterion plus two minor clinical criteria for diagnostic of neurocysticercosis. The major neuroimaging criterion was that all patients had CT-scans neuroimaging typical of parenchymal calcified nerocysticercosis. The clinical criteria were: (i) All pateints had seizures, the most common clinical manifestations suggestive of NCC; (ii) All individuals were coming and living in a cysticercosis-endemic area. Additionally, other causes of parenchymal calcification were excluded based in the neuroimaging caracteristics and previous medical history of the patients. Only two patients had neuropathological confirmation of neurocysticercosis. We do not perform biopsy or routine serologic tests or CSF test for neurocysticercosis in patients with cNCC (55).

### Ethics

The study was approved by the Research Ethics Committee of the HCPA/UFRGS (#2019-0043), and was conducted according to the principles of the Declaration of Helsinki.

### Statistical Analysis

Continuous variables were analyzed and as appropriated, by the Student t-test, Mann-Whitney U test, or ANOVA with Tukey's test, depending on the normality of data distribution. The Shapiro–Wilk test was used to test for normality. The Chi-squared or Fisher's exact test was used for categorical variables, and the results are expressed as odds ratio (OR) with 95% confidence interval (CI). All analyses were performed using SPSS software (IBM Corp. Released 2013. IBM SPSS Statistics for Windows, version 21.0. Armonk, NY: IBM Corp). The level of significance was set at *p* < 0.05.

## Results

**Figure 1:**
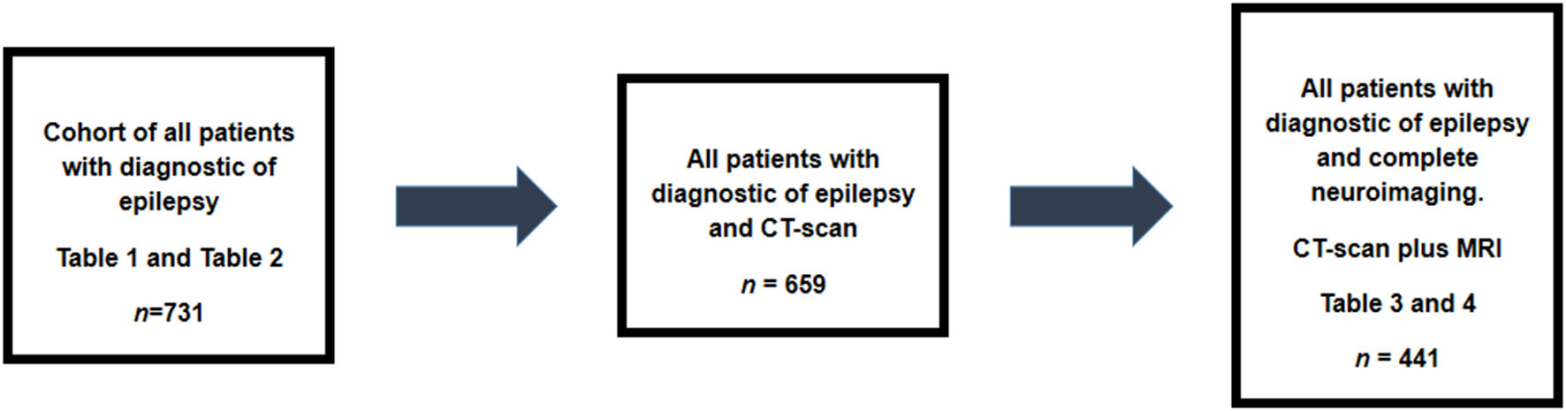
Design of the study. Data of all 731 patient were collected. From these, 659 patients had CT-scans and 42 of them had imaging compatible with cNCC. Complete neuroimaging was available for reviewing in 441patients. The Tables showing main analysis done are included in this figure making easy to follow the steps of this study.

**Figure 2:**
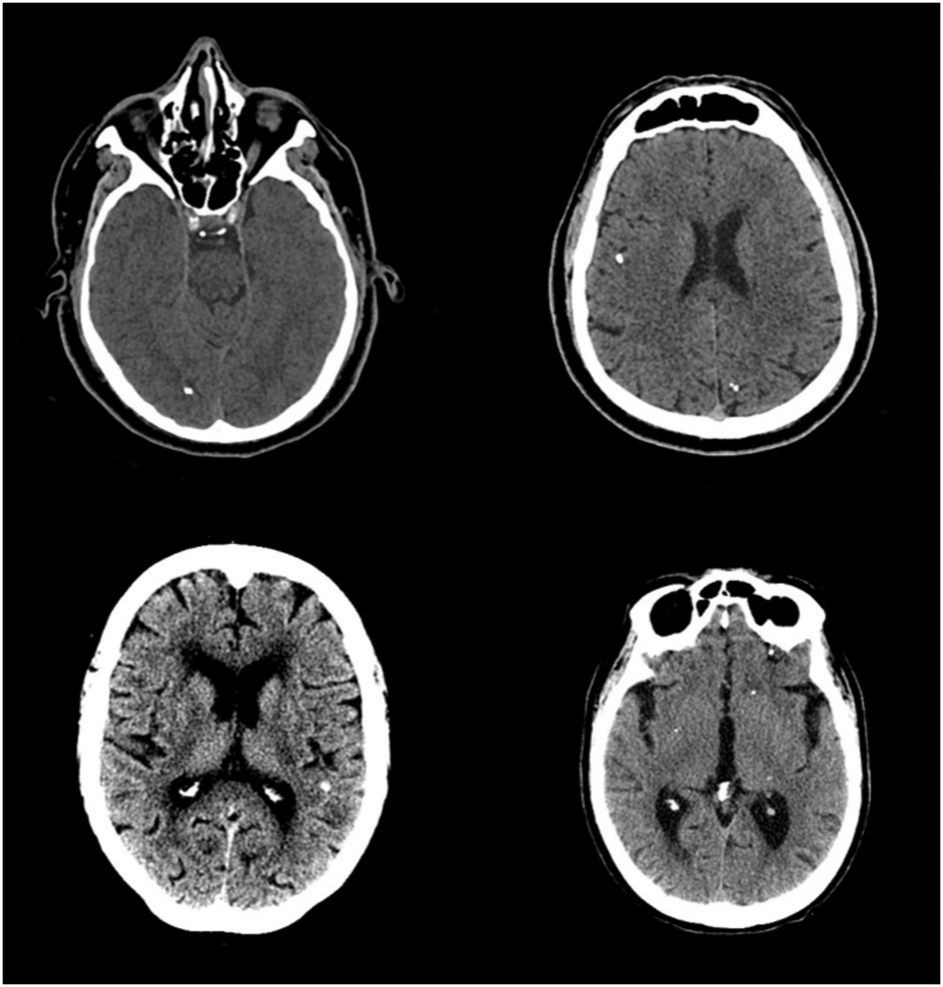
CT-scans of four different patients showing small calcifications (1–10 mm in diameter), single or multiple, located in brain parenchyma. These lesions are typically observed in patients with calcified neurocysticercosis and can be easily observed using CT-scan.

From 731 patients, 659 patients had CT-scan and 441 had both, CT-scan and MRI (Figure 1). Brazil is a country with limited resources for public health care or research and, unfortunately, complete neuroimaging for all the patients is not usually possible. Thus, as routine, patient with generalized epilepsy are not submitted to neuroimaging. Also, in general, patients with focal epilepsy whose cause can be diagnosed with CT-scan are not always submitted to MRI studies. However, we have MRI for all patients with cNCC for investigating the possibility of the association of hippocampal sclerosis in cNCC patients. Calcified lesions typical of NCC is present in Figure 2.

The demographic and clinical variables of the entire cohort are presented in Table 1. cNCC lesions were observed more frequently in women, occurring in 33 (78.6%) female patients, as opposed to 9 (21.4%) male patients. This was maintained also when only the 659 patients with CT-scan were analyzed (*n* = 33–78.6 vs. *n* = 09–21.4%, respectively; OR = 3.64;(95%CI = 1.71–7.69); *p* < 0.001, data not showed). cNCC also occurred significantly more often in patients who developed epilepsy later in life, in older patients and in patients who had a longer history of epilepsy. Also, cNCC was observed in patients with lower educational level.

**Table 1:** Demographic variables of all 731 patients.

**cNCC** **Variables**	**Total** ***n* = 731 (%)**	**Without cNCC** ***n* = 689 (%)**	**With cNCC** ***n* = 42 (%)**	**O.R. (95%CI)**	***p***
Sex					
Male	342 (46.8%)	333 (48.3%)	09 (21.4%)		
Female	389 (53.2%)	356 (51.7%)	33 (78.6%)	3.44 (1.64–7.69)	0.001*
Age	43.98 (16.54)	43.28 (16.48)	55.35 (13.06)	–	**<** 0.001*
Age at epilepsy Onset	17.03 (17.42)	16.68 (17.39)	22.88 (16.97)	–	0.025*
Time of epilepsy	26.94 (15.10)	26.60 (14.94)	32.46 (16.71)	–	0.015*
Years of school education					
≤ 4	326 (44.6%)	302 (43.8%)	24 (57.1%)		
5–8	244 (33.4%)	228 (33.1%)	16 (38.1%)		
9–12	132 (18.0%)	130 (18.9%)	02 (04.8%)		
> 12	029 (04.0%)	029 (04.2%)	00 (00.0%)	–	0.046*
Type of epilepsy					
Focal	630 (86.2%)	590 (85.6%)	40 (95.2%)		
Generalized	101 (13.8%)	99 (14.4%)	02 (04.8%)	3.45 (0.8–14.29)	0.104
Controlled seizures					
Yes	405 (55.4%)	385 (55.9%)	20 (47.6%)		
No	326 (44.6%)	304 (44.1%)	22 (52.4%)	1.39 (0.74–2.60)	0.296

cNCC, calcified neurocysticercosis; (*) bold = significant.

Table 2 presents the patients according to electroclinical syndromes and etiology. In our cohort, the etiology of epilepsy corresponded to the common causes of epilepsy. For example, a post-infectious cause of epilepsy other than cNCC was observed in 45 (6.2%) of 731 patients. Other causes of focal epilepsy were post-stroke epilepsy, observed in 36 (4.9%) patients, and epilepsy associated with vascular malformation, observed in 10 (1.4%) patients. Hypoxic-ischemic encephalopathy occurred in 83 (11.4%) patients and tumor-associated epilepsy in 27 (3.7%). Importantly for this study, 93 (12.7%) of the 731 patients had MTLE-HS.

**Table 2:** Patients according with electroclinical syndromes and epilepsy etiology.

**Electroclinical syndromes and epilepsy etiology**	**Number of patients (%)**
Mesial temporal lobe epilepsy with hippocampal sclerosis	93 (12.7%)
All other causes of epilepsy except MTLE-HS	
Hypoxic-ischemic encephalopathy	83 (11.4%)
Traumatic Brain Injury	47 (06.4%)
Infections, except cNCC	45 (06.2%)
Epilepsy with GTCS alone	44 (06.0%)
Stroke	36 (04.9%)
Juvenile Myoclonic Epilepsy	29 (04.0%)
Tumor	27 (03.7%)
cNCC without HS	17 (02.3%)
Lennox-Gastaut	12 (01.6%)
Vascular malformation	10 (01.4%)
Malformation of cortical development	02 (00.3%)
Miscellaneous	40 (05.5%)
Unknown	246 (33.7%)

*MTLE-HS, mesial temporal lobe epilepsy with hippocampal sclerosis; cNCC, calcified neurocysticercosis; HS, hippocampal sclerosis; GTCS, generalized tonic-clonic seizures*.

**Figure 3:**
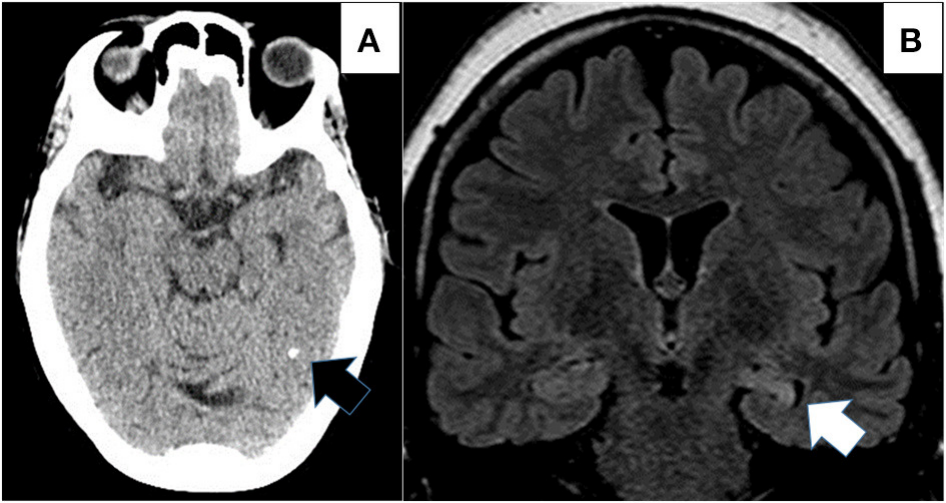
**(A)** CT-scan showing a single calcification (black arrow) highly suggestive of cNCC. **(B)** MRI FLAIR coronal imaging showing findings of hippocampal sclerosis (white arrow), typically observed in patients with mesial temporal lobe epilepsy associated with hippocampal sclerosis.

Table 3 presents clinical and demographic variables only for the patients that had complete neuroimaging (both, CT-scan and MRI). The results are similar to those presented in Table 1. Calcified NCC lesions occurred in 33 (78.6%) female patients, as opposed to 9 (21.4%) male patients (O.R. = 3.17; 95% C.I. = 1.48–6.80; *p* = 0.002). Figure 3 is a CT-scan showing a typical single cNCC lesion in (A) and in (B) MRI findings of hippocampal sclerosis in MTLE-HS. As observed for all cohort, in patients with complete neuroimaging, cNCC also occurred significantly more often in patients who developed epilepsy later in life, in older patients and in patients who had a longer history of epilepsy.

**Table 3:** Demographic variables—only patients with complete neuroimaging.

**cNCC** **Variables**	**Total *n =* 441 (%)**	**Without cNCC** ***n* = 399 (%)**	**With cNCC** ***n* = 42 (%)**	**O.R. (95%CI)**	***p***
Sex					
Male	194 (44.0)	185 (46.4)	09 (21.4)		
Female	247 (56.0)	214 (53.6)	33 (78.6)	3.17 (1.48–6.80)	0.002*
Age	44.02 (16.50)	42.83 (16.38)	55.36 (13.07)	–	**<** 0.001*
Age at epilepsy onset	17.41 (17.02)	16.84 (16.95)	22.89 (16.98)	–	0.028*
Time of epilepsy	26.61 (14.97)	25.99 (14.67)	32.47 (16.71)	–	0.008*
Years of school education					
≤ 4	202 (45.8%)	178 (44.6%)	24 (57.1%)		
5–8	132 (29.9%)	116 (29.1%)	16 (38.1%)		
9–12	88 (20.0%)	86 (21.6%)	02 (04.8%)		
> 12	19 (04.3%)	19 (04.8%)	00 (00.0%)	–	0.021*
Type of epilepsy					
Focal	402 (91.2%)	362 (90.7%)	40 (95.2%)		
Generalized	039 (8.8%)	37 (09.3%)	02 (04.8%)	0.49 (0.11–2.11)	0.564
Controlled seizures					
Yes	226 (51.2%)	206 (51.5%)	20 (47.6%)		
No	215 (48.8%)	193 (48.4%)	22 (52.4%)	1.17 (0.62–2.22)	0.631

cNCC, calcified neurocysticercosis; (*) bold = significant.

Table 4 shows characteristics of the 441 patients with complete neuroimaging (CT-scan plus MRI), divided according with presence of cNCC alone, MTLE-HS alone, MTLE-HS plus cNCC or other forms of epilepsy. Results confirm previous observations that cNCC occurred more often in female and older patients.

**Table 4:** Characteristics of patients with complete neuroimaging (*n* = 441), divided according with presence of cNCC alone, MTLE-HS alone, MTLE-HS plus cNCC and other forms of epilepsy.

**Groups** **variables**	**cNCC (*n =* 17)**	**MTLE-HS** **(*n =* 68)**	**MTLE-HS plus** **cNCC (*n =* 25)**	**Other** **(*n =* 331)**	***p***
Sex					
Male	04 (23.5%)	29 (42.6%)	05 (20.0%)	156 (47.1%)	
Female	13 (76.5%)	39 (57.4%)	20 (80.0%)	175 (52.9%)	0.018*
Age	51.9 (13.9)	50.8 (14.16)	57.7 (12.2)	41.2 (16.4)	**<** **0.001**
Age at epilepsy onset	22.8 (15.2)	15.2 (14.4)	22.9 (18.4)	17.2 (17.4)	0.135
Time of epilepsy	29.1 (18.4)	35.6 (13.3)	34.7 (15.4)	24.0 (13.9)	**<** **0.001**
Years of school education					
≤ 4	12 (70.6%)	34 (50.0%)	12 (48.0%)	144	
5–8	04 (23.5%)	20 (29.4%)	12 (48.0%)	96	
9–12	01 (05.9%)	12 (17.7%)	01 (04.0%)	74	
> 12	00 (00.0%)	02 (02.9%)	00 (00.0%)	17	0.101

cNCC, calcified neurocysticercosis; MTLE-HS, mesial temporal lobe epilepsy associated with hippocampal sclerosis. (*) bold = significant.

Table 5 shows that 42 patients (6.4%) of the of the 659 patients had cNCC. However, 25 (59.5%) of these patients had also MTLE-HS. cNCC was observed in only 17 (3.0%) of 566 patients with other types of epilepsy rather than MTLE-HS, a highly statistically significant difference; O.R. = 11.90, (95%CI = 6.10–23.26), *p* < 0.0001. The results were similar when considering only the 441 patients with complete neuroimaging (CT-scan AND MRI). In fact, when only these patients were analyzed alone, 93 (21.1%) of them showed MTLE-HS and cNCC was present in 25 of these 93 (26.9%). cNCC was observed in only 17 (4.9%) of the 348 patients with other types of epilepsy rather than MTLE-HS, with a highly statistically significant difference persisting (O.R. = 7.14; 95% CI = 3.67–13.89; *p* < 0.0001).

**Table 5:** MTLE-HS according with the presence or absence of neurocysticercosis.

			**All Patients with CT-scan = 659 patients**		
	**Total**	**Without cNCC**	**With cNCC**	**O.R. (95%CI)**	***p***
MTLE-HS	093 (14.1%)	068 (73.1%)	25 (26.9%)		
Other	566 (85.9%)	549 (97.0%)	17 (03.0%)	**11.90 (6.10–23.26)**	**<** 0.0001*
			Only Patients with complete neuroimaging (CT-scan and MRI) = 441 patients		
	**Total**	**Without cNCC**	**With cNCC**	**O.R. (95%CI)**	***p***
MTLE-HS	093 (21.1%)	068 (73.1%)	25 (26.9%)		
Other	348 (78.9%)	331 (95.1%)	17 (04.9%)	**07.14 (3.67–13.89)**	**<** 0.0001*

MTLE-HS, mesial temporal lobe epilepsy with hippocampal sclerosis; cNCC, calcified neurocysticercosis; (*) bold, significant. All patients with CT-scan showing cNCC had also MRI for hippocampal evaluation.

Regarding side of cNCC and side of hippocampal sclerosis, 20 (47.6%) from 42 patients with cNCC had a single calcified lesion and 22 (52.4%) had more than one calcification. From the 20 patients with a single cNCC, 11 patients had also neuroimaging findings of hippocampal sclerosis and clinical findings of MTLE-HS (ten patients had unilateral and one patient had bilateral hippocampal sclerosis). From these patients with unilateral hippocampal sclerosis, nine patients had unilateral hippocampal sclerosis with ipsilateral cNCC lesion (06 temporal, 02 occipital and 01 parietal) and one patient had a frontal cNCC lesion that was contralateral to the hippocampal sclerosis. Regarding EEG findings, all 11 patients with a single cNCC lesion plus MTLE-HS had EEG interictal abnormalities typical of MTLE-HS, compatible with neuroimaging findings of hippocampal sclerosis. Considering the 09 patients with a single cNCC and no MRI findings of hippocampal sclerosis, EEG was considered normal in two patients. In three patients with extra-temporal cNCC lesions, interictal EEG showed irritative zone over the temporal lobes. EEG showed extra-temporal interictal spikes in additional four patients. In three of them, these EEG findings were congruent with the cNCC lesional zone and one patient showed EEG with generalized 3.5 HZ spike and waves.

Considering the 22 patients with multiple calcifications (more than one calcification), fourteen had neuroimaging findings of hippocampal sclerosis (11 unilateral and 03 bilateral), with clinical and EEG findings compatible with MTLE-HS. Eight patients had no hippocampal MRI abnormalities. From these eight patients, EEG was normal in 02 patients, showed extra-temporal abnormalities in 3 of them and in 03 patients EEG showed only temporal abnormalities (01 unilaterally and 02 bilaterally).

## Discussion

In this study, cNCC was observed mainly in patients with MTLE-HS. Of 42 patients with cNCC, 25 also had HS. Calcified NCC was also observed in association with other forms of epilepsy, being a probable cause of focal epilepsy in 16 patients and probably an occasional finding in one patient. In our cohort, cNCC patients were older and with few years of formal education, indicating perhaps that NCC is decreasing as the sanitary conditions are improving and the population is becoming more urbanized. Interestingly, in our patients, cNCC was significantly more observed in women than in men. Our findings are also in line with previous reports showing that in patients a single cNCC lesion, the cNCC lesion is more often located in the same cerebral hemisphere that the hippocampal sclerosis, usually in the temporal lobe. This is interesting because is a finding perhaps suggesting that the cNCC and MTLE-HS association cannot be explained only by the presence of two unrelated but common disease affecting the same population (34, 36, 42, 45).

In last decades, an association between NCC and MTLE-HS has been observed in some patients (33, 34, 57). Initially, researchers speculated that this association might occur only by chance, as the two diseases are common forms of epilepsy, especially in developing countries (34, 56). However, if that were the case, it would be expected that cNCC lesions would be seen homogenously distributed across the different types of epilepsies. However, in the present study, there was an unexpectedly high prevalence of cNCC in patients with MTLE-HS. Other authors have described this association before. Velasco et al. (34), in a case-control study, found that cNCC was observed more frequently in patients with MTLE-HS than in patients with other forms of drug-resistant epilepsy undergoing surgical treatment. Taveira et al. (43), in another case-control study, observed that cNCC was more frequent in MTLE-HS than in other forms of epilepsies or in patients with headache. Oliveira et al., comparing MTLE-HS with primary generalized epilepsy and other forms of focal symptomatic epilepsy and after controlling for confounding factors and socioeconomic variables, observed an independent association between cNCC lesions and MTLE-HS (66). Although these studies came from different centers, all are specialized in epilepsy surgery and located in the state of São Paulo, Brazil. Thus, observations in other populations and other regions of the world are necessary in order to estimate the magnitude of the association of cNCC and MTLE-HS worldwide. In this venue, few other observations have been reported for other world regions. In general, the available studies are also confirming this association. These recent studies have been also exploring potential pathophysiological mechanisms that could explain the MTLE-HS-cNCC relationship, focusing on how NCC could cause or contribute to the development of hippocampal lesion and MTLE-HS, and thus exploring this question from the perspective of a cause-effect relationship (12, 36, 38, 44, 73). Rathore et al. (38) observed that patients with MTLE-HS and cNCC had a lower incidence of febrile seizures, older age at IPI, and more diffuse epileptiform discharges compared to patients with MTLE-HS alone, findings in line with some of our previous results (35, 40, 79). A possible cause-effect of NCC and MTLE-HS has also been suggested by some other authors (12, 74, 75, 77, 78).

At this point, perhaps there is sufficient evidence to support the view that cNCC might be highly associated with MTLE-HS in some populations. Therefore, it is time to question why such an association exists. For the reasons discussed above, the simple explanation that both diseases are common in the developing world and can be seen in the same patient might be true for some cases, but not for all of them (34, 45, 56). Thus, several authors have suggested that NCC may have a more direct role in the development of MTLE-HS or even cause HS. According to this hypothesis, NCC would be a type of CNS infection that could act as an IPI leading to hippocampal damage and MTLE-HS later in life due to inflammatory mechanisms or repetitive seizures. These two mechanisms are not mutually exclusive and both could contribute to the development of HS and MTLE-HS (33–45, 74, 75, 77, 78). Some case reports seem to point in this direction. However, observations of isolated cases or case series are not sufficient to confirm a cause-effect relationship between acquiring NCC and developing HS and MTLE-HS. The ideal confirmatory study would be a prospective study evaluating a large population in an endemic area for NCC and observing the eventual development of HS and MTLE-HS in these patients, similarly to the FABSTAT study (80). However, such studies cannot be performed due to the existence of effective treatments for NCC that could prevent epilepsy. Thus, observations in case series and animal models might provide the indirect evidences necessary to determine whether, how and how much NCC is related to MTLE-HS.

NCC is a known causative agent of epilepsy. However, the concept that NCC can cause or contribute to the development of MTLE-HS is relatively new. Some authors believe that this can be explained by the fact that the understanding of the physiopathogenesis of MTLE-HS is relatively recent and far for being complete. The IPI concept was developed in areas of the world where NCC was absent or rare. On the other hand, NCC occurs in world areas where medical research or medical resources for patient investigations are far less available as in the developed world. These two aspects might have contributed to a delay in observing an association and possible cause-effect relationship between NCC and MTLE-HS. In fact, to properly study such association, it is essential to have good quality clinical records, interictal and ictal EEG exams, CT-scans for better observation of cNCC lesions and detailed MRI to properly evaluate HS. This is perhaps the reason why the first observations about a possible association of NCC with MTLE-HS came from epilepsy surgery centers. At these centers, focal epilepsies must be extensively studied prior to epilepsy surgery, especially in the case of dual pathology, as might be the case of NCC and MTLE-HS. In fact, insights into a possible interaction between cNCC and MTLE-HS and possible mechanisms of association have occurred during these assessments and during the surgical decision-making process, so that initial reports on these matters have come from epilepsy surgical centers. Now, the association of cNCC and MTLE-HS has been also corroborated in other populations, as is the case for this study.

**Figure 4:**
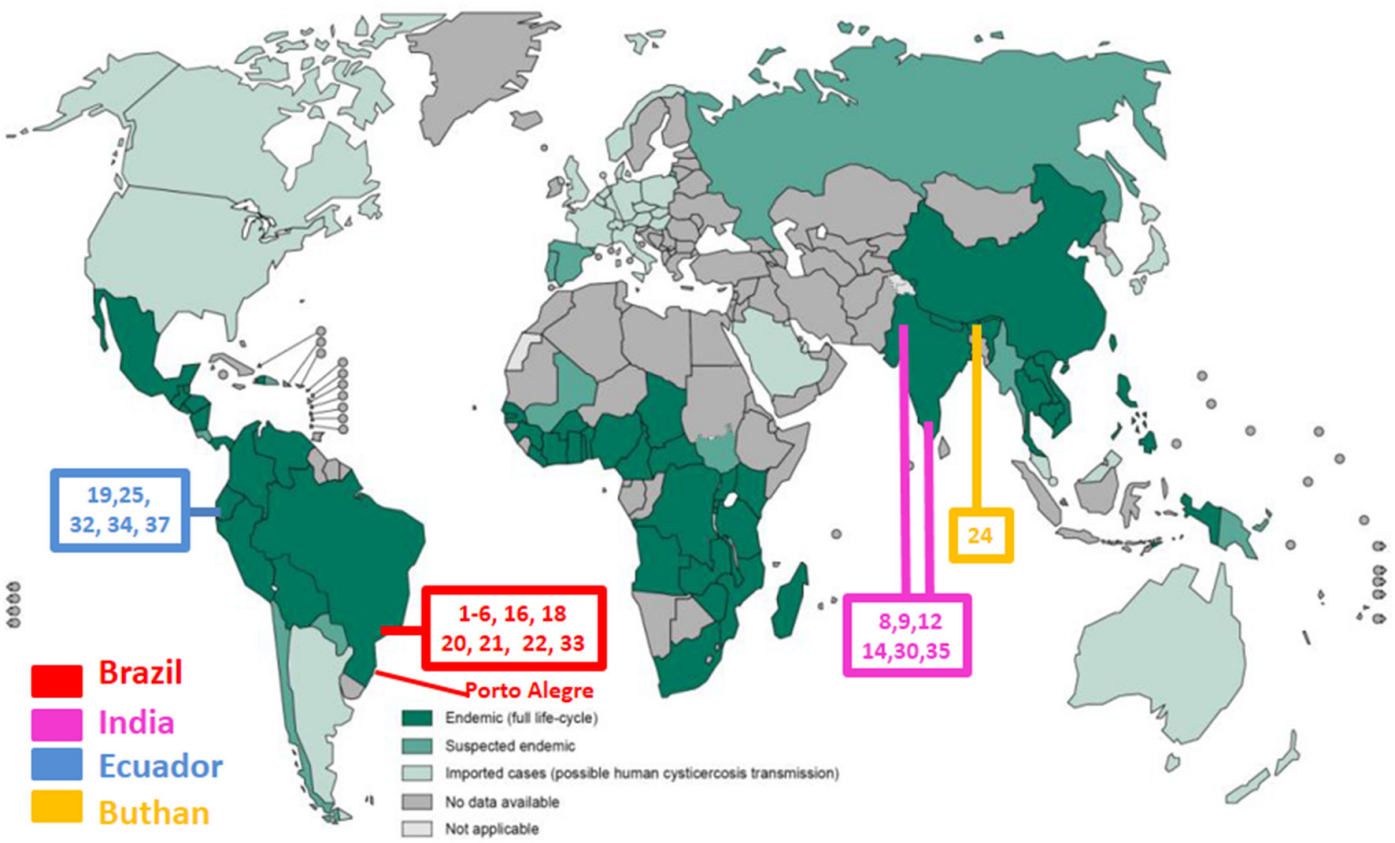
The map is showing countries at risk for neurocysticercosis in the world, according to World Health Organization (/WHO). at:http://apps.who.int/iris/bitstream/10665/153237/1/9789241508452_eng.pdf?ua = 1. [Last accessed November, 2021]. The map is also showing places were main of the original clinical work evaluating the association of neurocysticercosis and mesial temporal lobe epilepsy associates with hippocamapal sclerosis were done. Numbers are references to these works, as detailed in Table 6.

Table 6 shows main study published in MEDLINE that evaluate a possible association of MTLE-HS. It shows the type of the study and the population evaluated. These studies were obtained using “neurocysticercosis AND [hippocamp^*^ OR (temporal AND epilepsy)]” as the search strategy. This recover 83 studies, spanning from 1989 to 2021. Only 25 studies have clinical original data. The data are case reports, series of cases, case-control studies and evaluations of cohorts. Most of them are retrospective and evaluate the association of NCC and MTLE-HS studying patients with cNCC and MTLE-HS. Unfortunately, only few case-reports have documented a time relationship between the NCC infection and posterior development of the MTLE-HS. However, these case reports are very important because they highly suggest a cause-effect relationship between NCC infection and posterior development of MTLE-HS. Figure 4 represents a world map showing regions endemic for neurocysticercosis and places that have studies on the association of NCC and MTLE-HS. Very few researchers are conducing these studies. Most of these studies came from the same populations in Brazil (São Paulo State), Atahualpa, a coastal rural community in Ecuador and from few places in India. Considering the worldwide prevalence of NCC, and looking at this map, it becomes clear that much more studies, performed in many other populations and by other researches are necessary to understand the real impact of NCC in MTLE-HS in the world. That was one of the reasons that we performed this study in Porto Alegre, a city located more than one thousands of kilometers from São Paulo.

**Table 6:** Main studies on neurocysticercosis and hippocampal sclerosis.

**Author, year**	**Place**	**Type of study**	**Main conclusion**
**(1) Leite et al**. (**56**)	**Ribeirão Preto, Brazil**	**Case-control**	**MTLE-HS was associated with cNNC, but not causally**.
**(2) Kabayashi et al**. (**57**)	**Campinas, Brazil**	**Case report**	**NCC caused MTLE-HS**.
**(3) Wichert-Ana et al**. (**33**)	**Ribeirão Preto, Brazil**	**Case report**	**NCC caused MTLE-HS**.
**(4) Da Gama et al**. (**58**)	**Campinas, Brazil**	**Cross-sectional**	**MTLE-HS was associated with cNNC, but not causally**.
**(5) Da Silva et al**. (**59**)	**São Paulo, Brazil**	**Case report**	**NCC caused MTLE**.
**(6) Velasco et al**. (**34**)	**Ribeirão Preto, Brazil**	**Cross-sectional**	**MTLE-HS was associated with cNNC. This association may be causal or not**.
(7) Bianchin et al. (35)	São Paulo, Brazil	Letter	NCC may cause MTLE-HS.
**(8) Singla et al**. (**36**)	**Ludhiana, India**	**Case-series**	**NCC caused MTLE-HS in four patients**.
**(9) Chandra et al**. (**60**)	**New Delhi, India**	**Case-series**	**MTLE-HS was associated with cNNC. This association may be causal or not**.
(10) Bianchin et al. (61)	Ribeirão Preto, Brazil	Letter	NCC may cause or contribute with MTLE-HS.
(11) Bianchin et al. (37)	Ribeirão Preto, Brazil	Review	NCC may cause or contribute with MTLE-HS.
**(12) Rathoree et al**. (**38**)	**Kerala, India**	**Case-control**	**NCC causes or contributes with MTLE-HS**.
(13) Singh et al. (62)	Punjab, India	Review	NCC may cause MTLE-HS.
**(14) Rathoree et al**. (**63**)	**Kerala, India**	**Case-series**	**NCC may cause MTLE-HS**.
(15) Carpio et al. (64)	Cuenca, Ecuador	Review	More studies are needed.
**(16) Bianchin et al**. (**40**)	**Ribeirão Preto, Brazil**	**Cohort**	**NCC causes or contributes with MTLE-HS**.
(17) Singh et al. (65)	Punjab, India	Review	NCC may cause MTLE-HS.
**(18) Oliveira et al**. (**66**)	**São Paulo, Brazil**	**Case-control**	**NCC may cause MTLE-HS**.
**(19) Del Brutto et al**. (**67**)	**Atahualpa, Ecuador**	**Cross-sectional**	**NCC and hippocampal atrophy association**.
**(20) Bianchin et al**. (**42**)	**Ribeirão Preto, Brazil**	**Cohort**	**NCC causes or contributes with MTLE-HS**.
**(21) Meguins et al**. (**68**)	**São Paulo, Brazil**	**Cohort**	**NCC worse pre-existing MTLE-HS**.
**(22) de Oliveira et al**. (**43**)	**Campinas, Brazil**	**Case-control**	**NCC may cause MTLE-HS**.
(23) Del Brutto et al. (44)	Guayaquil, Ecuador	Review	NCC may cause MTLE-HS.
**(24) Brizzi et al**. (**69**)	**Thimphu, Bhutan**	**Cross-sectional**	**NCC was not associated with MTLE-HS**.
**(25) Del Brutto et al**. (**70**)	**Atahualpa, Ecuador**	**Case-control**	**NCC and hippocampal atrophy association**.
(26) Bianchin et al. (45)	Porto Alegre, Brazil	Review	NCC may cause or contribute with MTLE-HS.
(27) Escalaya et al. (71)	Lima, Peru	Review	NCC may cause MTLE-HS.
(28) Duque et al. (13)	Lima, Peru	Review	NCC may cause MTLE-HS.
(29) Ramantani et al. (17)	Vogtareuth, Germany	Review	NCC may cause MTLE-HS.
**(30) Aulakh** (**72**)	**Chandigarh, India**	**Case report**	**NCC caused MTLE-HS**.
(31) Singh et al. (73)	London, UK	Review	NCC may cause MTLE-HS.
**(32) Issa et al**. (**74**)	**Atahualpa, Ecuador**	**Case-control**	**NCC and hippocampal atrophy association**.
**(33) JamaAntónio et al**. (**75**)	**Campinas, Brazil**	**Case-control**	**NCC and hippocampal atrophy association**.
**(34) Del Brutto et al**. (**76**)	**Atahualpa, Ecuador**	**Case-control**	**NCC and hippocampal atrophy association**.
**(35) Mhatre et al**. (**77**)	**Bangalore, India**	**Cross-sectional**	**NCC may cause MTLE-HS**.
(36) Herrick et al. (12)	Chicago, Illinois	Review	NCC may cause MTLE-HS.
**(37) Del Brutto et al**. (**78**)	**Atahualpa, Ecuador**	**Cohort**	**NCC may cause hippocampal atrophy**.

*NCC, neurocysticercosis; cNCC, calcified neurocysticercosis; MTLE-HS, mesial temporal lobe epilepsy associated with hippocampal sclerosis. In bold are represented human studies with original data*.

Due to the several limitations of observational clinical studies as discussed above, preclinical studies and experimental models are very important to better understand the association between NCC and MTLE-HS. In fact, some possible mechanism involved in this association perhaps can only be inferred from animal models. *Taenia crassiceps* infection in mice is a model of human cysticercosis. In this model, *Taenia crassiceps* infection leads to great damage and apoptosis in the hippocampus, mainly in the dentate gyrus, hillus, CA1, CA2, CA3 and neighboring brain regions of the animal. Some of these findings parallel those observed in HS in MTLE-HS patients (81, 82). It is also interesting to note that, in these animal models, there is also gender-specific differential expression of cytokines in specific regions of the brain, including the hippocampus (83), a finding that may help to explain the gender differences observed in patients with cNCC and MTLE-HS. Moreover, the observations that women can develop more aggressive and inflammatory forms of NCC (84) might further suggest that inflammatory mechanisms are possibly involved in the association of cNCC and MTLE-HS and it may explain why this association is more common in women. It may also explain why we found more women with cNCC and epilepsy than men in our study, a finding also observed by other authors (43). In addition, in animal models, there are also reports of abnormal brain electrical activity, immunological abnormalities, hormonal and gene expression alterations, and neurochemical changes that could influence epileptogenicity in the hippocampus, hypothetically favoring the development of hippocampal damage and MTLE-HS in patients with NCC (85–90).

This study has limitations. It is a retrospective study in which it was included an entire cohort of patients with epilepsy, although neuroimaging studies were not available for all of the patients. However, most patients with focal epilepsy had neuroimaging. Furthermore, only the patients with available complete neuroimaging, both CT-scan and MRI were also evaluated and the results remained similar and conclusions were still the same from those observed in the entire cohort. Also, the diagnosis of cNCC was based on neuroimaging findings of cNCC plus clinical criteria. Only two patients had anatomopathological confirmation of NCC. Blood or cerebrospinal fluid test for neurocysticercosis were not done in these patients. These tests are not done as routine in patients with cNCC or other forms of epilepsy in our outpatient clinic. These are also limitations of our study. Moreover, this study was done at a referral hospital and thus it perhaps have analyzed a more selected group of patient that may not be representative of the entire population, even if it is a public hospital open to all patients and not restricted to refractory surgical patients only. Due to its retrospective nature, it was not possible to establish a cause-effect relationship between NCC infection and the development of MTLE-HS and therefore some of our conclusions regarding this matter are speculative. However, our study has some strengths that also need to be recognized. It was conducted on a population never studied for these aspects before. Since it is a study of a cohort of patients with epilepsy, it was possible to observe two interesting aspects of the association between cNCC and MTLE-HS that could not be observed if this had been a case-control study: the high prevalence of cNCC and MTLE-HS association and the difference in sex prevalence of this association. These observations are important because the former might suggest a cause-effect relationship between NCC infection and later development or worsening of MTLE-HS. The sex differences might suggest some possible mechanisms for this association, once there are evidence that women are prone to develop more important inflammatory response to NCC than men (84).

When considering the findings of this work as a whole, this study might leads to four main conclusions. First, in endemic areas for NCC, patients need to be evaluated with both CT-scans, which can show cNCC better than MRI, and MRI for the assessment of HS. Evaluating patients with NCC with a CT-scan alone is inadequate because CT-scan may not reveal imaging abnormalities of mesial structures, including hippocampal sclerosis. Second, as expected, some patients might have epilepsy directly related to a neurocysticercosis associated lesional zone, although this was surprisingly observed in fewer patients than previously imagined by us at the beginning of the study. Third, other forms of epilepsy can also be seen in patients with cNCC just by chance, and in these patients, epilepsy seems not directly related to cNCC. Forth, cNCC might be very commonly associated with MTLE-HS, an observation that agrees with the hypothesis that NCC can contribute to or directly cause MTLE-HS in many patients. Thus, perhaps these observations might contribute to a better evaluation of patients with cNCC and epilepsy and a better understanding of the impact of NCC on the development of MTLE-HS in endemic NCC regions and of some characteristics of this association. Given the worldwide prevalence of NCC and the relatively few studies in this field, it is clear that more studies are necessary to better understand how NCC contributes to epilepsy and the real dimension of the association between NCC and MTLE-HS, two of the most common causes of epilepsy worldwide.

## Data Availability Statement

The raw data supporting the conclusions of this article will be made available by the authors, without undue reservation.

## Ethics Statement

The studies involving human participants were reviewed and approved by Ethics Committee of Hospital de Clinicas de Porto Alegre. The patients/participants provided their written informed consent to participate in this study.

## Author Contributions

TS and MB: conception and design of the work, drafting the work, and revising the manuscript. TS and RB: acquisition of data for the work. TS, RB, JBr, JBi, and MB: analysis, interpretation of data, revision, and approval of the final version of the manuscript. All authors contributed to the article and approved the submitted version.

## Funding

The author MB is supported by Conselho Nacional de Desenvolvimento Científico e Tecnológico (CNPq) #312683/2018-9.

## Conflict of Interest

The authors declare that the research was conducted in the absence of any commercial or financial relationships that could be construed as a potential conflict of interest.

## Publisher's Note

All claims expressed in this article are solely those of the authors and do not necessarily represent those of their affiliated organizations, or those of the publisher, the editors and the reviewers. Any product that may be evaluated in this article, or claim that may be made by its manufacturer, is not guaranteed or endorsed by the publisher.
